# The vitamin E isoforms α-tocopherol and γ-tocopherol have opposite associations with spirometric parameters: the CARDIA study

**DOI:** 10.1186/1465-9921-15-31

**Published:** 2014-03-15

**Authors:** Michelle E Marchese, Rajesh Kumar, Laura A Colangelo, Pedro C Avila, David R Jacobs, Myron Gross, Akshay Sood, Kiang Liu, Joan M Cook-Mills

**Affiliations:** 1 Division of Allergy and Immunology, Feinberg School of Medicine, Northwestern University, McGaw M304, 240 E. Huron, Chicago, IL, 60611, USA; 2 The Ann and Robert H. Lurie Children’s Hospital of Chicago, Feinberg School of Medicine, Northwestern University, Chicago, IL 60611, USA; 3 Department of Preventive Medicine, Feinberg School of Medicine, Northwestern University, Chicago, IL 60611, USA; 4 Division of Epidemiology and Community Health, University of Minnesota, School of Public Health, Minneapolis, MN 55454, USA; 5 Department of Nutrition, School of Medicine, University of Oslo, Oslo, Norway; 6 Laboratory Medicine and Pathology, University of Minnesota, Minneapolis, MN 55455, USA; 7 Division of Pulmonary and Critical Care Medicine, University of New Mexico, Albuquerque, NM 87131, USA

**Keywords:** α-tocopherol, γ-tocopherol, FEV_1_, FVC, Human

## Abstract

**Background:**

Clinical studies of the associations of vitamin E with lung function have reported conflicting results. However, these reports primarily examine the α-tocopherol isoform of vitamin E and have not included the isoform γ-tocopherol which we recently demonstrated in vitro opposes the function of α-tocopherol. We previously demonstrated, in vitro and in animal studies, that the vitamin E isoform α-tocopherol protects, but the isoform γ-tocopherol promotes lung inflammation and airway hyperresponsiveness.

**Methods:**

To translate these findings to humans, we conducted analysis of 4526 adults in the Coronary Artery Risk Development in Young Adults (CARDIA) multi-center cohort with available spirometry and tocopherol data in blacks and whites. Spirometry was obtained at years 0, 5, 10, and 20 and serum tocopherol was from years 0, 7 and 15 of CARDIA.

**Results:**

In cross-sectional regression analysis at year 0, higher γ-tocopherol associated with lower FEV_1_ (p = 0.03 in blacks and p = 0.01 in all participants) and FVC (p = 0.01 in blacks, p = 0.05 in whites, and p = 0.005 in all participants), whereas higher α-tocopherol associated with higher FVC (p = 0.04 in blacks and whites and p = 0.01 in all participants). In the lowest quartile of α-tocopherol, higher γ-tocopherol associated with a lower FEV_1_ (p = 0.05 in blacks and p = 0.02 in all participants). In contrast, in the lowest quartile of γ-tocopherol, higher α-tocopherol associated with a higher FEV_1_ (p = 0.03) in blacks. Serum γ-tocopherol >10 μM was associated with a 175–545 ml lower FEV_1_ and FVC at ages 21–55 years.

**Conclusion:**

Increasing serum concentrations of γ-tocopherol were associated with lower FEV1 or FVC, whereas increasing serum concentrations of α-tocopherol was associated with higher FEV1 or FVC. Based on the prevalence of serum γ-tocopherol >10 μM in adults in CARDIA and the adult U.S. population in the 2011 census, we expect that the lower FEV_1_ and FVC at these concentrations of serum γ-tocopherol occur in up to 4.5 million adults in the population.

## Background

There are seemingly conflicting results in clinical studies with vitamin E. As we discuss in a recent perspective [1] and recent reviews [2-4], conflicting outcomes in clinical studies and differences in prevalence of allergic disease among countries may, at least in part, reflect differences in the serum levels of γ-tocopherol. There are four tocopherol isoforms of vitamin E: α-tocopherol, γ-tocopherol, β-tocopherol and δ-tocopherol. The vitamin E isoforms, α-tocopherol and γ-tocopherol are the most abundant in diet and tissues, and α-tocopherol is the most studied. α-tocopherol and γ-tocopherol differ by one methyl group and, at equal molar concentrations, have a relatively similar capacity to scavenge reactive oxygen species (ROS) [5,6]. In contrast to α-tocopherol, γ-tocopherol also reacts with reactive nitrogen species [7] and may be beneficial for inflammation with increases in reactive nitrogen species such as neutrophilic inflammation [8,9].

Although the western diet is abundant in γ-tocopherol, α-tocopherol is about 10 fold higher than γ-tocopherol in tissues due primarily to the preferential transfer of α-tocopherol to lipid particles by liver α-tocopherol transfer protein [10] and due to a higher rate of production of γ-tocopherol metabolites for excretion [11]. In the U.S., the mean adult serum γ-tocopherol is about 5–7 μM, whereas European and other countries have a 2–6 fold lower serum γ-tocopherol concentration [1-4]. These differences in serum γ-tocopherol are consistent with European diets rich in olive oil, which is low in γ-tocopherol, and American diets rich in soy oil, which is high in γ-tocopherol [1]. In contrast, the average serum concentrations of α-tocopherol are similar among these populations [1-4].

We have reported that, in mice, α-tocopherol supplementation improves and γ-tocopherol supplementation worsens eosinophilic lung inflammation and airway hyperresponsiveness [12]. When treated with both α-tocopherol and γ-tocopherol, γ-tocopherol ablates the benefit of α-tocopherol [12]. In mechanistic studies, we demonstrated that α-tocopherol inhibits and γ-tocopherol increases leukocyte recruitment and the activation of protein kinase Cα (PKCα) during leukocyte recruitment [12-14]. Moreover, we demonstrated that these tocopherols directly bind to PKCα and that α-tocopherol is an antagonist and γ-tocopherol is an agonist of PKCα [14]. However, it is not known whether these tocopherol isoforms have opposing associations with lung spirometry in humans.

We hypothesized that in humans, serum α-tocopherol and γ-tocopherol have opposing associations with spirometric parameters and that there is an association of tocopherols with these parameters when the concentration of the opposing tocopherol is low and causing the least competing opposing effects [1]. To test these hypotheses, we analyzed a database with existing data for α-tocopherol, γ-tocopherol and spirometry in a large cohort of 5114 participants with 20 years follow-up.

## Results

### Participant description at year 0 examination

We determined whether there are opposing associations of α-tocopherol and γ-tocopherol with the spirometry parameters FEV_1_, FVC, and FEV_1_/FVC in humans using the CARDIA study with 20 years participant follow-up and existing data for spirometry and tocopherol isoforms. Participant characteristics are in Table 1. By design for CARDIA, the study had similar numbers of males and females of each race. The mean serum α-tocopherol concentration was lower and the mean γ-tocopherol concentration was higher in black compared to white participants at study year 0 (Table 2). This is consistent with previous reports [15,16]. Due to racial differences in tocopherol concentrations, and because there are known race-based differences in spirometric parameters [17], we stratified the dataset by race. Also, because females and males are known to differ in spirometric parameters [17], the analysis included adjustments for gender.

**Table 1:** Participant characteristics (year 0 of the CARDIA study)

	**Blacks**	**Whites**	**Total**	**P value**
Number of participants	2289 (50.6%)	2237 (49.4%)	4526	
Male	1032 (49.4%)	1057 (50.6%)	2089	0.14
Female	1257 (51.6%)	1180 (48.4%)	2437	
Mean age, years (SD)	24.3 (3.8)	25.5 (3.4)	4526	<0.0001
Current smoker	782 (34.2%)	611 (27.3%)	1393	<0.0001
Past smoker	210 (9.2%)	397 (17.8%)	607	
Non-smoker	1297 (56.7%)	1229 (54.9%)	2526	
Ever reported asthma	493 (21.5%)	427 (19.1%)	920	0.04
BMI, kg/m^2^ (SD)	25.48 (5.63)	23.71 (4.05)	4526	<0.0001
FEV1 (liters) [2SD Range]	3.28 [1.90-4.66]	3.83 [2.30-5.36]	4526	<0.0001
% predicted FEV1 (SD)	98.2 (12.7)	97.6 (10.7)	4526	0.06
FVC (liters) [2 SD Range]	3.93 [2.21-5.66]	4.68 [2.67-6.69]	4526	<0.0001
% predicted FVC (SD)	101.0 (12.4)	100 (10.6)	4526	0.002
FEV_1_/FVC (%)	83.72	82.36	4526	<0.0001
Median household income (study year 5)	$25,000-34,999	$35,000-49,999	3806	<0.0001
Years of education (SD)	13.03 (1.82)	14.59 (2.38)	4526	<0.0001

**Table 2:** Serum α-tocopherol (α-T) and γ-tocopherol (γ-T) levels (year 0 of the CARDIA study)

	**Blacks**					**Whites**					
	**N**	**Mean (μM)**	**SD (μM)**	**SE (μM)**	**Range (μM)**	**N**	**Mean (μM)**	**SD (μM)**	**SE (μM)**	**Range (μM)**	**P value**
α-T	2289	19.71	5.28	0.11	7.57-59.39	2237	22.68	6.69	0.14	5.73- 84.91	<0.0001
γ-T	2289	5.11	2.40	0.05	0.14- 67.41	2237	4.64	1.97	0.04	0.00- 27.02	<0.0001

### γ-tocopherol and α-tocopherol have opposing associations with spirometry parameters at year 0 examination

The cross-sectional, multivariable linear regressions examined the association of α-tocopherol with spirometric parameters and γ-tocopherol with spirometric parameters. The data are presented as difference in parameter analyzed per 10 μM tocopherol, as previously described for association studies with tocopherol [18]. In the cross-sectional analysis, higher serum γ-tocopherol was significantly associated with lower FEV_1_ and FVC in blacks (Table 3). In whites and in analysis of all participants, there was a significant association of higher γ-tocopherol with lower FVC (Table 3).

**Table 3:** The association of α-tocopherol (α-T) and γ-tocopherol (γ-T) with lung spirometry (year 0 of the CARDIA study)

	**Lung spirometry**	**BLACKS**^**a**^					**WHITES**^**a**^					**ALL PARTICIPANTS**^**b**^				
		**N**	**β**^**c**^	**SE**	**95% confidence interval**	**P value**	**N**	**β**	**SE**	**95% confidence interval**	**P value**	**N**	**β**	**SE**	**95% confidence interval**	**P value**
α-T		2289					2237					4526				
	FEV_1_		27.6	17.3	-6.3 to 61.6	**0.11**^**#**^		5.5	13.7	-21.4 to 32.4	0.69		11.1	10.8	-10.1 to 32.3	0.30
	FVC		40.4	19.7	1.9 to 78.9	0.04*		33.4	16.0	2.1 to 64.7	0.04*		31.3	12.5	6.8 to 55.8	0.01*
	FEV_1_/FVC		-0.1	0.3	-0.6 to 0.4	0.78		-0.4	0.2	-0.8 to -0.0	0.03*		-0.3	0.2	-0.6 to 0.0	**0.07**^**#**^
γ-T		2289					2237					4526				
	FEV_1_		-80.0	37.7	-154.0 to -6.0	0.03*		-78.0	47.1	-170.3 to 14.3	**0.10**^**#**^		-73.7	29.5	-131.6 to -15.8	0.01*
	FVC		-106.7	42.8	-190.7 to -22.7	0.01*		-107.8	54.8	-215.3 to -0.4	0.05*		-97.0	34.1	-163.9 to -30.0	0.005*
	FEV_1_/FVC		0.3	0.5	-0.8 to 1.4	0.59		0.2	0.7	-1.1 to 1.5	0.74		0.2	0.4	-0.6 to 1.1	0.58

(Linear regression analysis at year 0 of the CARDIA study).

^a^Adjustments: center, age, age^2^, height, height^2^, sex, BMI, smoking status and ever asthma.

^b^Adjustments: center, age, age^2^, height, height^2^, sex, BMI, smoking status and ever asthma, race.

^c^β is the difference in mL of FEV1, mL of FVC or % FEV1/FVC per 10 μM tocopherol.

*and boldface, significant; ^#^and boldface, trend.

In contrast, to the inverse association of γ-tocopherol with spirometry, α-tocopherol was positively associated with FVC, suggesting opposing effects of the two tocopherol isoforms in blacks, whites, and all participants (Table 3). There was a positive association between α-tocopherol and FEV_1_ in black participants and in all participants and then, in whites, there was a trend for a positive association (Table 3). In whites, the FEV_1_/FVC was inversely associated with increasing serum α-tocopherol, although the beta was very small (Table 3). In these analyses for Table 3, the other tocopherol isoform is present at various concentrations with potentially opposing functions. Similar opposing associations of the tocopherol isoforms with spirometry were observed when the data analyses included adjustments for the other tocopherol isoform (Additional file 1: Table S1).

### Association of tocopherol isoforms with spirometry when the opposing tocopherol isoform is within its lowest quartile

The data in Table 3 suggested that the tocopherol isoforms have opposing associations with spirometry. Furthermore, in our mechanistic studies in vitro and in animals, γ-tocopherol potently increases inflammation and lung hyperresponsiveness when the α-tocopherol tissue concentration is low but this effect of γ-tocopherol is ablated when the α-tocopherol tissue concentration is elevated through supplementation [12-14]. Therefore, we hypothesized that there would be a significant association of tocopherols with FEV_1_ when the concentration of the opposing tocopherol was low, causing the least competing opposing effects. For this analysis, we used quartile analysis rather than α-tocopherol/γ-tocopherol ratios because ratios would be the same when both tocopherol isoforms are low and when both tocopherol isoforms are high and because we previously reported in mechanistic animal studies that there are dose dependent effects of tocopherol isoforms on lung inflammation [12,13]. Consistent with our hypothesis, in blacks at the lowest quartile of α-tocopherol, there was a significant negative association of FEV_1_ with increasing γ-tocopherol (Table 4A). At all quartile concentrations of α-tocopherol with the exception of quartile 2 in blacks for FEV_1_, the direction of FEV_1_ and FVC was decreasing with increasing units of γ-tocopherol (Table 4). In whites, at the lowest quartile of α-tocopherol, there was a trend for a negative association of FEV_1_ with increasing γ-tocopherol (Table 4B). For analysis of all participants (Table 4C), the negative beta for FEV_1_ with γ-tocopherol was greatest at the lowest quartile of α-tocopherol and the negative beta for FVC with γ-tocopherol was greatest at the lowest two quartiles of α-tocopherol (Table 4C).

**Table 4:** Association of γ-T with lung spirometry (within quartiles of α-tocopherol) at year 0 of CARDIA

**Quartiles of α-T**	**N**	**Difference in FEV**_**1 **_**per 10 μM γ-T**	**P value**	**Difference in FVC per 10 μM γ-T**	**P value**	**Difference in FEV**_**1**_**/FVC (%) per 10 μM γ-T**	**P value**
**(μM)**		**β**		**β**		**β**	
**A**	**BLACKS**						
5.73-17.21	596	-207.3	0.05*	-174.8	0.16	-1.0	0.55
17.22-20.11	522	8.2	0.94	-119.7	0.32	2.4	0.10
20.12-23.80	407	-103.4	0.38	-158.6	0.25	0.8	0.58
23.81-84.80	271	-103.9	0.06	-81.3	0.22	-0.8	0.29
**B**	**WHITES**						
5.73-17.21	317	-282.8	**0.10**^**#**^	-265.7	0.17	-1.2	0.60
17.22-20.10	389	-195.0	0.19	-271.3	0.14	0.7	0.71
20.11-23.80	500	-80.7	0.44	-42.3	0.73	-1.2	0.42
23.81-84.80	604	-80.1	0.25	-164.1	0.05*	0.9	0.34
**C**	**ALL PARTICIPANTS**						
5.73-17.21	913	-208.3	0.02*	-171.3	**0.11**^**#**^	-1.1	0.42
17.22-20.10	911	-58.7	0.50	-175.1	**0.09**^**#**^	2.0	**0.09**^**#**^
20.11-23.80	907	-82.4	0.29	-82.4	0.37	-0.3	0.77
23.81-84.80	875	-95.1	0.02*	-107.0	0.03*	-0.2	0.73

Year 0 of CARDIA: Adjustments: center, age, age^2^, height, height^2^, sex, BMI, smoking status.

*and boldface, significant; ^#^and boldface, trend.

Conversely and consistent with our hypothesis, at the lowest quartile of γ-tocopherol, there was a significant positive association of FEV_1_ and a trend for a positive association of FVC with increasing α-tocopherol in blacks (Table 5A). In whites, at the lowest quartile of γ-tocopherol, there was no association for α-tocopherol with FEV_1_ and FVC (Table 5B). For analysis of all participants, there was also no association for α-tocopherol with FEV_1_ and FVC (Table 5C). These data suggest that, for blacks and whites, associations for α-tocopherol with spirometry may be influenced by environmental or genetic differences that are not known.

**Table 5:** Association of α-T with lung spirometry (within quartiles of γ-tocopherol) at year 0 of CARDIA

**Quartiles of γ-T**	**N**	**Difference in FEV**_**1 **_**per 10 μM α-T**	**P value**	**Difference in FVC per 10 μM α-T**	**P value**	**Difference in FEV**_**1**_**/FVC (%) per 10 μM α-T**	**P value**
**(μM)**		**β**		**β**		**β**	
**A**	**BLACKS**						
0.00-3.59	375	73.8	0.03*	62.3	**0.11**^**#**^	0.6	0.20
3.60-4.69	432	-55.7	0.29	1.0	0.99	-1.3	0.05*
4.70-5.99	442	60.7	0.24	37.8	0.52	0.7	0.33
6.00-68.35	547	25.2	0.49	43.8	0.31	-0.3	0.55
**B**	**WHITES**						
0.00-3.59	538	-13.1	0.53	19.6	0.44	-0.6	0.04*
3.60-4.69	451	27.3	0.44	-18.1	0.65	-0.2	0.64
4.70-5.99	463	10.2	0.79	22.1	0.64	-0.1	0.80
6.00-68.35	358	37.0	0.34	84.4	0.05*	-0.7	0.18
**C**	**ALL PARTICIPANTS**						
0.00-3.59	913	11.8	0.51	29.9	0.16	-21.7	0.39
3.60-4.69	883	-33.8	0.24	-12.8	0.70	-0.5	0.20
4.70-5.99	905	24.9	0.42	17.9	0.63	0.3	0.51
6.00-68.35	905	20.7	0.43	50.7	**0.10**^**#**^	-0.5	0.16

Nonasthmatics, Year 0 of CARDIA (Adjustments: center, age, age^2^, height, height^2^, sex, BMI, smoking status).

*and boldface, significant; ^#^and boldface, trend.

### Modeling average γ-tocopherol concentrations for the USA and European/Asian countries reveals a significantly lower FEV_1_ and FVC in participants with highly-elevated γ-tocopherol

**Figure 1:**
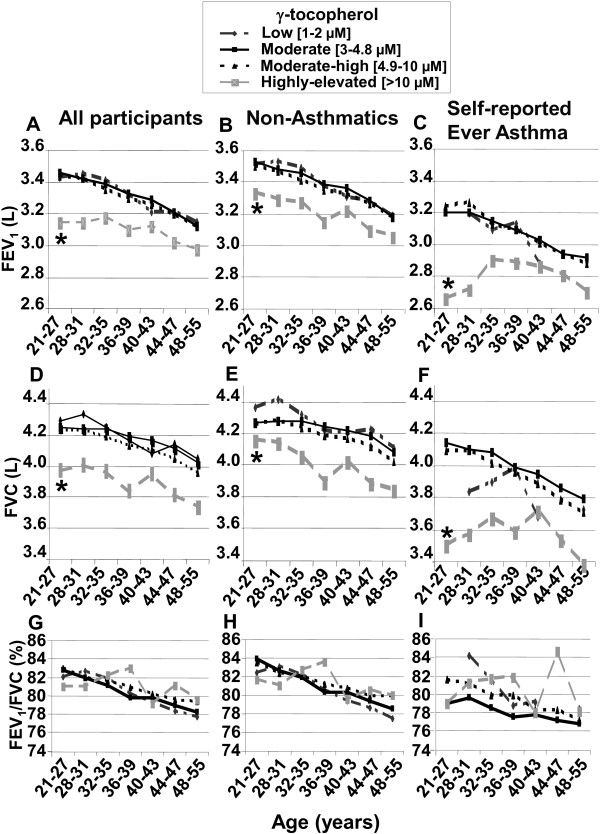
**Highly-elevated γ-tocopherol associates with reduced FEV**_**1 **_**and FVC at ages 21–55.** We a priori defined γ-tocopherol groups to model the average Western European/Asian γ-tocopherol levels (1–2 μM) (Additional file 2: Table S2) [4] and to model the average U.S.A. γ-tocopherol levels (3–4.8 μM and 4.9-10 μM) (Table 2 and Additional file 2: Table S2) [1-4]. We also defined a priori a γ-tocopherol group as >10 μM to model the 5-fold higher γ-tocopherol observed in our animal studies [19]. In study year 5, the four γ-tocopherol categories represented from lowest to highest γ-tocopherol groups of CARDIA: 36 (1.1%), 1291 (38.0%), 2015 (59.3%), and 58 (1.7%) participants. To examine the association of γ-tocopherol with FEV_1_, FVC and FEV1/FVC as a function of participant age, we used generalized estimating equations (GEE) to analyze spirometry and tocopherol as described in the Methods and Additional file 3: Methods. For GEE models, included were the following variables: center, race, exam year, height, height^2^, sex, BMI, smoking status, asthma group, average α-tocopherol concentration, 3 dummy variables representing the 4 γ-tocopherol groups, and the interactions of the four γ-tocopherol groups with age. The number of readings used for analysis for low, moderate, moderate-high, and highly-elevated γ-tocopherol groups were: **(A, D, G)** 93, 3493, 5381 and 160, respectively, for all participants; **(B, E, H)** 80, 2696, 4281, and 123, respectively, for non-asthmatics; and **(C, F, I)** 13, 797, 1100 and 37, respectively, for self-reported ever asthmatics. A missing data point in **C**, **F** and **I** at age 21–27 in the low γ-tocopherol group occurred because the participants at this age did not have this level of γ-tocopherol in the self-reported asthmatics. Nevertheless, for the self-reported asthmatics, the high γ-tocopherol group at age 21–27 was significantly different than the groups with moderate levels of γ-tocopherol. *, p < 0.05 compared to the other groups at 21–27 years old.

Given the difference in γ-tocopherol concentrations in Americans versus Western Europeans and Asians (Additional file 1: Table S1) [1,4] and the lung hyperreponsiveness promoting effect of a 5-fold increase in γ-tocopherol in our animal models [12,13], we determined whether a 5-fold higher γ-tocopherol associates with lower spirometry parameters in the CARDIA participants at ages 21–55 (Figure 1). For the analysis, we defined a priori a γ-tocopherol group to model the average Western European plasma γ-tocopherol levels and two γ-tocopherol groups to model the average USA plasma γ-tocopherol levels. The average Western European γ-tocopherol levels are 1–2 μM γ-tocopherol (Additional file 2: Table S2) [1] and therefore the group that models the average Western European plasma γ-tocopherol is 1–2 μM γ-tocopherol. The average USA γ-tocopherol levels in Table 2 and in previous reports (Additional file 2: Table S2) are 4–7 μM γ-tocopherol [19]. To model the average USA γ-tocopherol levels, two groups were defined a priori as moderate (3–4.8 μM, the average published for NHANES in the U. S.A. [19]) and moderate-high (4.9-10 μM, [1-4]). In addition, because a 5-fold higher γ-tocopherol in our animal studies reduced lung responsiveness, another group at >10 μM γ-tocopherol was defined a priori to model 5-fold higher γ-tocopherol than the average European plasma γ-tocopherol group (Figure 1).

Analysis was performed separately for non-asthmatics and for the participants with self-reported ever having asthma, because in Table 1, 20% of the participants reported ever having asthma and because our previous studies showed that γ-tocopherol reduced lung function of mice with experimental asthma [4,12,13]. In this analysis for participants at ages 21–55, we used spirometry from study years 5, 10, 20 and 25 and the tocopherol concentrations from study years 0, 7 and 15. We addressed the data limitation of lack of concordant spirometry and tocopherol data after year 0 of CARDIA by analyzing with GEE and averaging the CARDIA year 0, 7 and year 15 serum γ-tocopherol concentrations for each individual study participant because individuals tended to maintain their tocopherol relative positions over 15 years as analyzed by covariance of slope and intercept parameters (described in detail in the Manuscript Additional file 3: Methods). Also, the average of the three tocopherol measures reflects the long term exposure of the tocopherol. To limit the potential effects of opposing functions of very high α-tocopherol on spirometric parameters [13], and because it was determined in the cross-sectional analysis at CARDIA study year 0 that there were opposing effects of α-tocopherol and γ-tocopherol, it was determined a priori to include participants with a serum α-tocopherol level less than 37.1 μM because it is two standard deviations above the mean concentration of serum α-tocopherol in the 20–39 year age group from the U.S. National Health and Nutrition Examination Survey (NHANES) [19]. In addition, the average plasma α-tocopherol concentrations are about the same among several countries (Additional file 2: Table S2) [1,4]. After exclusions primarily for individuals who were missing data for tocopherol, spirometry or covariate at follow-up years (n = 623) and exclusion of a few individuals with very high serum α-tocopherol levels (over 37.1 μM) (n = 146), there were 3757 participants from years 5, 10, 20 and 25 of CARDIA for analysis. Because our analysis of this CARDIA cohort at year 0 revealed similar associations of α-tocopherol and γ-tocopherol in both races, we did not stratify the data by race in this analysis, but instead we adjusted for race to maintain the sample size of the highest and lowest γ-tocopherol groups. A GEE analysis was also performed with percent predicted values for FEV_1_ and the statistical outcomes were the same (data not shown).

Statistical data for Figure 1 is as follows: For 21–27 year old CARDIA participants, the FEV_1_ for the highly-elevated γ-tocopherol group is lower than the low, moderate, and moderate-high γ-tocopherol groups by: A) 317 mL (p = 0.006), 284 mL (p = 0.0003), and 263 mL (p = 0.0008), respectively, for all CARDIA participants (non-asthmatics and those who self-reported that they ever had asthma); B) 255 mL (p = 0.04), 213 mL (p = 0.01) and 175 mL (p = 0.03), respectively, for non-asthmatics; and C) 460 mL (p = 0.08), 475 mL (p < 0.009), and 517 mL (p = 0.004), respectively, for self-reported ever asthmatics. For 21–27 year old CARDIA participants, the FVC for the highly-elevated γ-tocopherol group is lower than the low, moderate, and moderate-high γ-tocopherol groups by: D) 322 mL (p = 0.01), 263 mL (p = 0.004), and 254 mL (p = 0.005), respectively, for all participants; E) 263 mL (p = 0.05), 160 mL (p = 0.08) and 156 mL (p = 0.08), respectively, for non-asthmatics; and F) 357 mL (p = 0.22), 568 mL (p = 0.009) and 545 mL (p = 0.01), respectively, for self-reporting ever asthmatics. For FEV_1,_ the tests for interaction of the continuous age variable for the highly-elevated γ-tocopherol group compared to the low, moderate and moderate-high γ-tocopherol groups were as follows: A) p = 0.07, p = 0.12, and p = 0.11, respectively, in all participants; B) p = 0.23, p = 0.45 and p = 0.58, respectively, in the non-asthmatics; and C) p = 0.21, p = 0.10 and p = 0.05, respectively, in those with “ever asthma”. For FVC_,_ the tests for interaction of the continuous age variable for the highly-elevated γ-tocopherol group compared to the low, moderate and moderate-high γ-tocopherol groups were as follows: D) p = 0.60, p = 0.89, and p = 0.63, respectively, for all participants; E) p = 0.98, p = 0.25 and p = 0.65, respectively, for non-asthmatics; and F) p = 0.53, p = 0.31 and p = 0.22, respectively, for those reporting “ever asthma”.

Figure 1 presents data for all CARDIA participants and also stratifies results by asthma status. For the 21–27 year olds, FEV_1_ for the highly-elevated γ-tocopherol group was significantly lower than the low, moderate, or moderate-high γ-tocopherol groups that modeled the average European/Asian and USA plasma γ-tocopherol levels (Figure 1A-C). Similarly, in the 21–27 year olds, FVC of the highly-elevated γ-tocopherol group was significantly lower than the other γ-tocopherol groups (Figure 1D-F). For ages 21–55, there was no difference in slopes of FEV1 and FVC (NS test for interaction with age). Therefore, because FEV_1_ and FVC for the highly-elevated γ-tocopherol group was significantly lower than the other γ-tocopherol groups at the age 21–27 and then there was no difference among the slopes of the lines to age 55, FEV_1_ and FVC of the highly-elevated γ-tocopherol group was lower than the other γ-tocopherol groups (Figure 1A-F). The analysis of FEV_1_/FVC resulted in no significant differences among the γ-tocopherol groups (Figure 1G-I).

## Discussion

In CARDIA, α-tocopherol positively associated with and γ-tocopherol negatively associated with spirometric parameters. Moreover, in the tocopherol isoform quartile analysis, the associations of tocopherols with FEV_1_ were most evident when the concentration of the opposing tocopherol was low and causing the least competing opposing effects. In modeling average γ-tocopherol concentrations for the USA and European/Asian countries, a 5-fold higher γ-tocopherol associated with reduced FEV_1_ and FVC in the 21–27 year olds and this reduction was sustained to age 55.

Based on our cross-sectional results for blacks with -80 mL FEV_1_/10 μM γ-tocopherol in Table 3, the study participants at two standard deviations above the mean of serum γ-tocopherol have about a 77 mL lower FEV_1_, compared with the participants at two standard deviations below the mean serum γ-tocopherol. Similarly, study participants with the highest range of γ-tocopherol would have about a 100 mL lower FVC. These reductions in spirometric parameters are even higher for participants with the lowest quartile of α-tocopherol because in these participants, there is a -207 to -282 mL FEV_1_/10 μM γ-tocopherol for blacks and white, respectively, in Table 4.

There was also a difference in spirometry parameters over the range of α-tocopherol concentrations. The two standard deviations above and below the average α-tocopherol in black CARDIA participants was 9.15-30.27 μM at year 0 of CARDIA. The cross-sectional analysis indicates that each 10 μM higher α-tocopherol associates with 40.4 mL higher FVC in blacks. Therefore, a study participant at the higher range of α-tocopherol (30.27 μM) should have about 85 mL greater FVC than an individual at the lower level. This is similar in magnitude to another report indicating that an estimated intake of 2 mg/day of vitamin E in foods in the United Kingdom associates with a 39 mL increase in FEV_1_[20]. These magnitudes are also similar to that reported for vitamin C. Vitamin C intake (100 mg/day) is reported to associate with a 22–42 mL increase in FEV_1_ and a 25–80 ml increase in FVC [21].

A strength of this study is the cohort size for analysis of effects of tocopherols with the least amount of the opposing tocopherol. The limitation that in years 7–25, spirometry data and plasma for tocopherol analysis were not collected at the same time was addressed using GEE analysis. The GEE analysis indicates that at ages 21–55, highly-elevated levels of serum γ-tocopherol associated with significantly lower FEV_1_ and FVC. Furthermore, since the slopes in spirometry with age were not different, it suggests that the reduction in FEV_1_ and FVC associated with tocopherol may be achieved before age 21. This finding is consistent with potential vitamin E regulation of human lung development because maternal α-tocopherol positively associates with fetal length and with FEV_1_ in 5 year old children in a report in the United Kingdom [22,23]. The γ-tocopherol-associated decreases in FEV_1_ and FVC before age 21 may occur during lung responses to environmental pollutants, allergens, or infections because tocopherols can directly regulate PKC [3,4,12,13]. Tocopherols have an effect on plateau spirometry parameters. This effect may be relevant for spirometry parameters later in life. Furthermore, based on the about 2% prevalence with highly-elevated (>10 μM) serum γ-tocopherol in adults in CARDIA and the U.S. 2011 census of adults, we expect that the 350–570 milliliters lower FEV_1_ or FVC occurs in up to 4.5 million adults in the population. This is a clinically significant volume because it corresponds to 9 to 15 years of decline in lung function with aging [24].

The γ-tocopherol association with reduced FEV_1_ and FVC was exacerbated in the asthmatic group, resulting in participants having 350–570 mL lower FEV_1_ or FVC in the highly-elevated γ-tocopherol group as compared to the low to moderate γ-tocopherol groups at ages 21–27. This 10 to 17% decrease in FEV_1_ is similar to the 5-10% reduction in FEV_1_ reported for other environmental factors. For example, individuals with occupational allergen exposure have a 5-8% decrease in FEV_1_ and this decrease is associated with dyspnea, chest tightness, chronic bronchitis, and chronic cough [25]. It is also reported that responders to particulate matter have a 2 to 6% decrease in FEV_1_[26], responders to cold or exercise have a 5 to 11% decrease in FEV_1_[27] and responders to house dust mite or dog/cat dander have a 2-8% decrease in FEV_1_[28].

## Conclusion

In summary, our study is the first to report that in humans, there are opposing associations for α-tocopherol and γ-tocopherol with spirometric parameters. α-tocopherol associates with higher spirometric parameters and γ-tocopherol associates with lower spirometric parameters. Moreover, our analysis suggests that the opposing effects of an isoform of tocopherol occur in participants with the lowest range of the opposing isoform of tocopherol. These associations of tocopherol isoforms with spirometric parameters in humans are consistent with the mechanistic studies of opposing functions for α-tocopherol and γ-tocopherol regulation of lung function in mice and cellular signaling [4,12,14].

## Methods

### Cohort description

**Figure 2:**
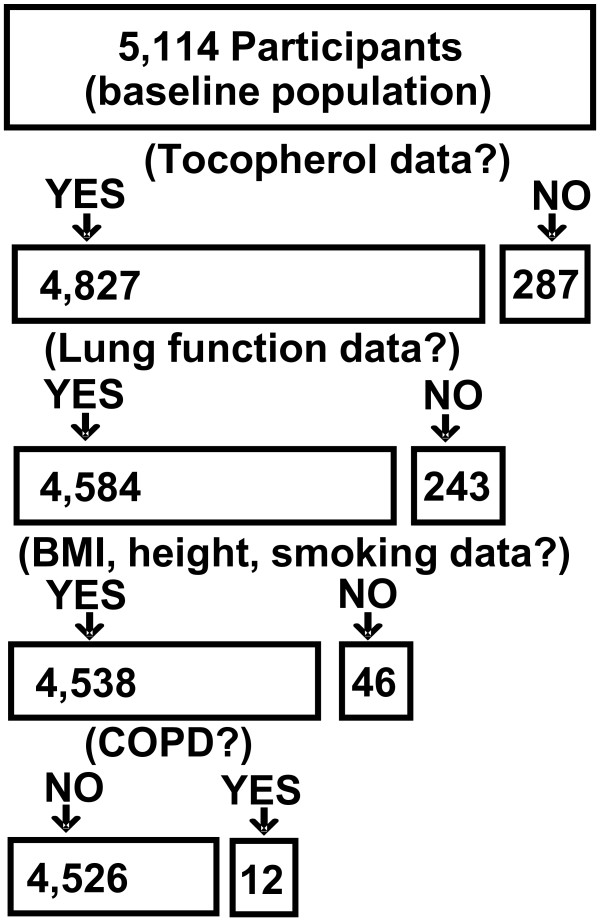
**Participant flow chart.** Of the 5115 participants recruited into CARDIA, 1 dropped out. Of the 5114 CARDIA remaining participants, 4827 participated in YALTA and had year 0 tocopherol measurements in the CARDIA database. Of the 4827, participants were excluded from the analysis if they were missing data for spirometry at year 0 (n = 243), missing data for smoking, body mass index, or height (n = 46) or self-reported having chronic obstructive pulmonary disease (COPD) (n = 12) (Figure 2). After these exclusions, there were 4526 participants in the analytic sample. Of these, 920 study participants self-reported as ever having asthma and/or being treated for asthma at any of the 7 visits (Table 1). For our analysis, there were 4,526 participants after excluding participants with COPD or excluding participants that lacked data for tocopherol levels, spirometry, BMI, height or smoking.

CARDIA is a multi-center longitudinal cohort study designed to examine the risk factors for cardiovascular disease in young adults enrolled at 18–30 years [29-31]. By design, the study enrolled 5,114 participants in equal subgroups of race (black or white), and gender. Participants were assessed at year 0 (1985–1986) and years 2, 5, 7, 10, 15, and 20. Young Adult Longitudinal Trends in Antioxidants (YALTA), an ancillary CARDIA study, measured serum tocopherols from blood samples of fasting participants in years 0, 7, and 15 of the study [32,33]. After exclusions in Figure 2, there were 4,526 participants.

### Spirometry

The spirometry measurements FEV_1_ and FVC were at years 0, 2, 5, 10, and 20 as described in the Additional file 3. American Thoracic Society (ATS)/European Respiratory Society (ERS) guidelines were followed to ensure quality control and testing procedures [34].

### Tocopherol analysis

Tocopherols at years 0, 7, and 15 of the study were extracted from the serum and analyzed by high pressure liquid chromatography (HPLC) [35] as described in the Additional file 3.

### Statistical analyses

Details of each statistical analysis are in the Manuscript Additional file 3. Briefly, multivariable linear regressions were performed at study year 0 to determine the association between tocopherol concentrations and spirometry. Due to significant differences in tocopherol concentrations (p < 0.0001) by race, data were either stratified by race or adjusted for race as indicated in the tables and figures. To analyze associations of opposing effects of tocopherol isoforms, the association of each isoform was analyzed by strata based on quartiles of the opposing tocopherol using the SAS (Cary, NC, USA) GLM procedure.

To examine the association of γ-tocopherol with spirometry as a function of participant age, we used generalized estimating equations (GEE) as described in the Additional file 3 and figure legend. We used GEE analysis for FEV_1_, FVC and FEV_1_/FVC as a function of the age groups for presentation in the figure and also did analysis treating age as a continuous variable as described in the Additional file 3. The GEE analysis was also performed with percent predicted values for FEV_1_ and the statistical outcomes were the same (data not shown).

## Abbreviations

CARDIA: Coronary Artery Risk Development in Young Adults; COPD: Chronic obstructive pulmonary disease; FEV1: Forced expiratory volume in 1 second; FVC: Forced vital capacity; GEE: Generalized estimating equations; HPLC: High pressure liquid chromatography; NHANES: U.S. National Health and Nutrition Examination Survey; YALTA: Young Adult Longitudinal Trends in Antioxidants ancillary study.

## Competing interests

The authors declare that they have no competing interests.

## Authors’ contributions

MEM in Joan Cook-Mills’ research laboratory participated in design, statistical analysis and manuscript preparation. RK participated in analytical study design, clinical interpretations and manuscript preparation. LC is the statistician who performed the statistical analysis and participated in preparation of statistical methods section of manuscript. PA participated in design of clinical parameters for analysis. DJ and MG are the Principal Investigators of the YALTA study; they acquired the participant serum samples and analyzed tocopherols in them; they and AS participated in critical review and interpretation of the manuscript. KL participated in design of statistical analysis and manuscript review. JMC-M conceived of the study design and participated in statistical analysis, interpretations, and manuscript preparation. All authors read and approved the final manuscript.

## Supplementary Material

Additional file 1: Table S1 The association of α-tocopherol (α-T) and γ-tocopherol (γ-T) with lung spirometry with adjustment for other tocopherol isoform. (Linear regression analysis at year 0 of the CARDIA study). Click here for file

Additional file 2: Table S2 Plasma γ-tocopherol (γT) and α-tocopherol (αT) in several countries. Click here for file

Additional file 3 Detailed Methods. Click here for file
