# Supplementary material for: The vitamin E isoforms α-tocopherol and γ-tocopherol have opposite associations with spirometric parameters: the CARDIA study
Source: Respir Res. 2014 Mar 15;15(1):31. doi: 10.1186/1465-9921-15-31 (PMC4003816; doi:10.1186/1465-9921-15-31)
Supplement: Additional file 1: Table S1 — The association of α-tocopherol (α-T) and γ-tocopherol (γ-T) with lung spirometry with adjustment for other tocopherol isoform. (Linear regression analysis at year 0 of the CARDIA study). [file 1465-9921-15-31-S1.ppt]

## Slide 1
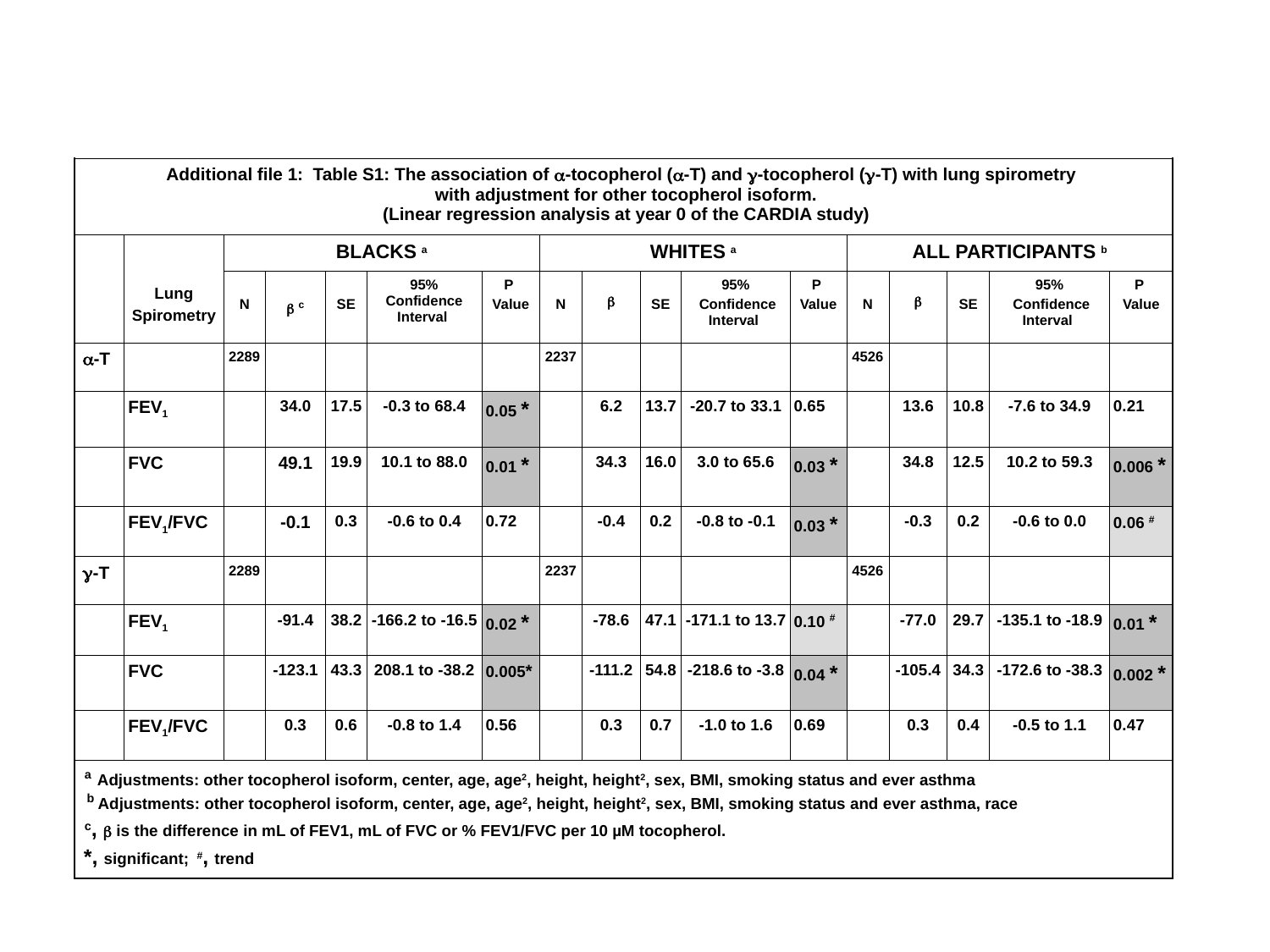

| Additional file 1: Table S1: The association of α-tocopherol (α-T) and γ-tocopherol (γ-T) with lung spirometry with adjustment for other tocopherol isoform. (Linear regression analysis at year 0 of the CARDIA study) | | | | | | | | | | | | | | | | |
| --- | --- | --- | --- | --- | --- | --- | --- | --- | --- | --- | --- | --- | --- | --- | --- | --- |
| | Lung Spirometry | BLACKS a | | | | | WHITES a | | | | | ALL PARTICIPANTS b | | | | |
| | | N | β c | SE | 95% Confidence Interval | P Value | N |  | SE | 95% Confidence Interval | P Value | N |  | SE | 95% Confidence Interval | P Value |
| α-T | | 2289 | | | | | 2237 | | | | | 4526 | | | | |
| | FEV1 | | 34.0 | 17.5 | -0.3 to 68.4 | 0.05 \* | | 6.2 | 13.7 | -20.7 to 33.1 | 0.65 | | 13.6 | 10.8 | -7.6 to 34.9 | 0.21 |
| | FVC | | 49.1 | 19.9 | 10.1 to 88.0 | 0.01 \* | | 34.3 | 16.0 | 3.0 to 65.6 | 0.03 \* | | 34.8 | 12.5 | 10.2 to 59.3 | 0.006 \* |
| | FEV1/FVC | | -0.1 | 0.3 | -0.6 to 0.4 | 0.72 | | -0.4 | 0.2 | -0.8 to -0.1 | 0.03 \* | | -0.3 | 0.2 | -0.6 to 0.0 | 0.06 # |
| γ-T | | 2289 | | | | | 2237 | | | | | 4526 | | | | |
| | FEV1 | | -91.4 | 38.2 | -166.2 to -16.5 | 0.02 \* | | -78.6 | 47.1 | -171.1 to 13.7 | 0.10 # | | -77.0 | 29.7 | -135.1 to -18.9 | 0.01 \* |
| | FVC | | -123.1 | 43.3 | 208.1 to -38.2 | 0.005\* | | -111.2 | 54.8 | -218.6 to -3.8 | 0.04 \* | | -105.4 | 34.3 | -172.6 to -38.3 | 0.002 \* |
| | FEV1/FVC | | 0.3 | 0.6 | -0.8 to 1.4 | 0.56 | | 0.3 | 0.7 | -1.0 to 1.6 | 0.69 | | 0.3 | 0.4 | -0.5 to 1.1 | 0.47 |
| a Adjustments: other tocopherol isoform, center, age, age2, height, height2, sex, BMI, smoking status and ever asthma b Adjustments: other tocopherol isoform, center, age, age2, height, height2, sex, BMI, smoking status and ever asthma, race c, β is the difference in mL of FEV1, mL of FVC or % FEV1/FVC per 10 µM tocopherol. \*, significant; #, trend | | | | | | | | | | | | | | | | |
