# Supplementary material for: The vitamin E isoforms α-tocopherol and γ-tocopherol have opposite associations with spirometric parameters: the CARDIA study
Source: Respir Res. 2014 Mar 15;15(1):31. doi: 10.1186/1465-9921-15-31 (PMC4003816; doi:10.1186/1465-9921-15-31)
Supplement: Additional file 2: Table S2 — Plasma γ-tocopherol (γT) and α-tocopherol (αT) in several countries. [file 1465-9921-15-31-S2.ppt]

## Slide 1
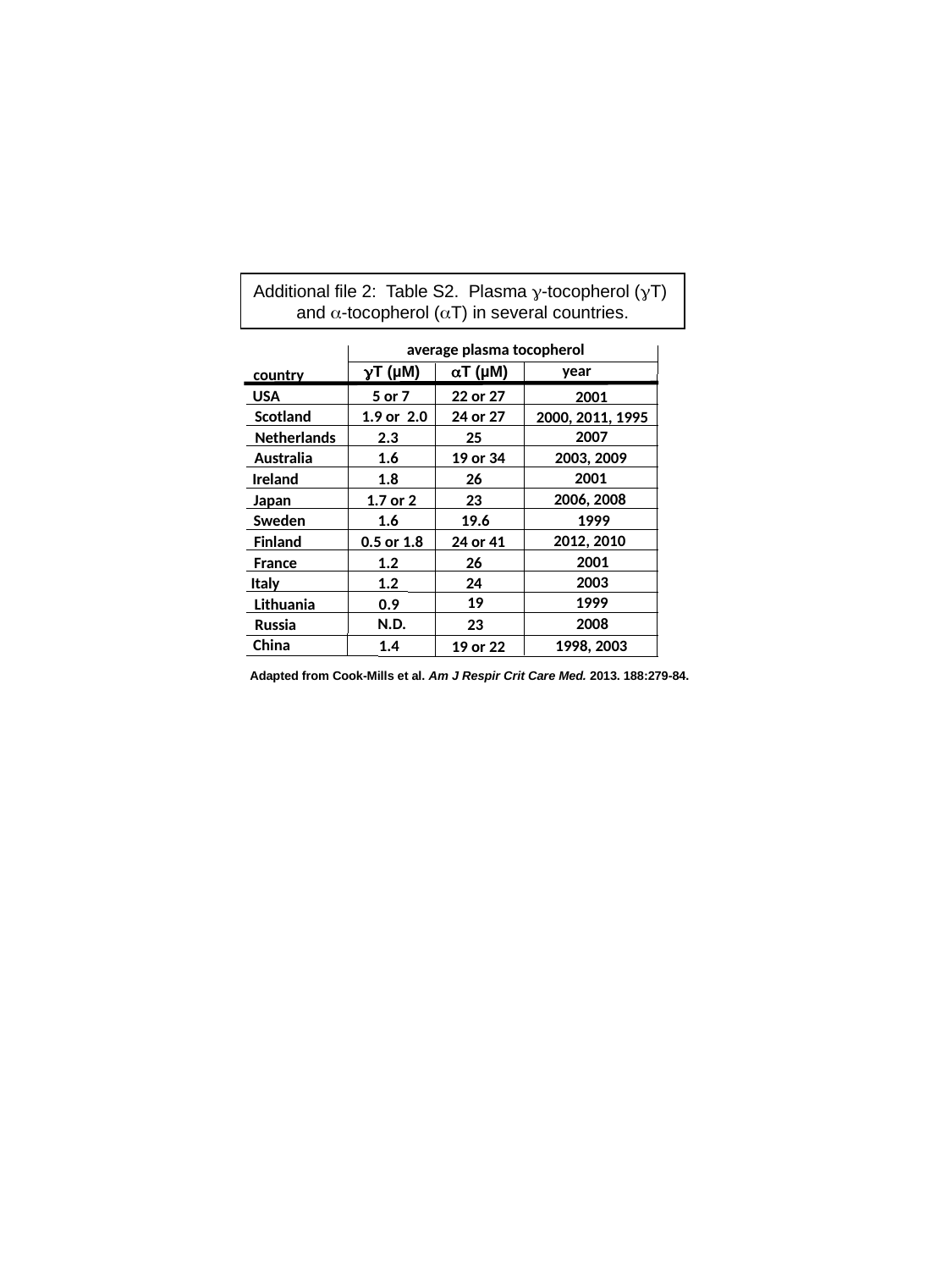

Additional file 2: Table S2. Plasma γ-tocopherol (γT)
and α-tocopherol (αT) in several countries.
average plasma tocopherol
γT (µM)
αT (µM)
year
country
2001
USA
5 or 7
22 or 27
2000, 2011, 1995
Scotland
1.9 or 2.0
24 or 27
2007
Netherlands
2.3
25
2003, 2009
Australia
1.6
19 or 34
2001
Ireland
1.8
26
2006, 2008
Japan
1.7 or 2
23
1999
Sweden
1.6
19.6
2012, 2010
Finland
0.5 or 1.8
24 or 41
2001
France
1.2
26
2003
Italy
1.2
24
1999
19
Lithuania
0.9
2008
N.D.
Russia
23
1998, 2003
China
1.4
19 or 22
Adapted from Cook-Mills et al. Am J Respir Crit Care Med. 2013. 188:279-84.
