# Supplementary material for: The vitamin E isoforms α-tocopherol and γ-tocopherol have opposite associations with spirometric parameters: the CARDIA study
Source: Respir Res. 2014 Mar 15;15(1):31. doi: 10.1186/1465-9921-15-31 (PMC4003816; doi:10.1186/1465-9921-15-31)
Supplement: Additional file 3 — Detailed Methods. [file 1465-9921-15-31-S3.doc]

**Additional file 3**

**Detailed Methods.**

# Spirometry.

Measurements were at years 0, 2, 5, 10, and 20. From year 0 through year 10, spirometry was measured with a Collins Survey 8-I water-sealed spirometer and an Eagle II Microprocessor (Warren E. Collins, Inc., Braintree, MA). At year 20, spirometry was measured with a dry-rolling OMI spirometer (Viasys, Loma Linda, CA). Twenty five volunteers at the LDS Hospital (Salt Lake City, UT) took part in a comparability study: the average difference between the Collins and OMI spirometers were 6 mL for forced vital capacity (FVC) and 21 mL for forced expiratory volume in 1 second (FEV_1_). Accuracy was determined using the Pulmonary Waveform Generator (MH Custom Design and Manufacturing, Midvale, UT), a computer-run spirometry simulator (accuracy of ±0.5%). American Thoracic Society (ATS)/European Respiratory Society (ERS) guidelines were followed to ensure quality control and testing procedures .

**Tocopherol Analysis.**

Blood samples were taken from fasting participants, and serum was isolated by centrifugation and stored at -70ºC . The serum concentrations of α-tocopherol and γ-tocopherol were measured by high pressure liquid chromatography (HPLC) at the Molecular Epidemiology and Biomarker Research Laboratory (University of Minnesota, Minneapolis) . The HPLC assay was modified from the Bieri et al method to measure tocopherols by adding 0.015% diisopropylethylamine to the HPLC solvent to aid in analyte recovery. Tocol was added as an internal standard. Tocopherols were detected at a 292 nm absorbance channel. Calibration was performed following the Craft et al method and samples were handled according to the standards described by Gross et al . Blood draws for tocopherol analysis were collected on years 0, 7 and 15 of CARDIA. Thus, tocopherol analysis only coincided with spirometry at year 0. Quality-control procedures included routine analysis of plasma and serum control pools containing high and low concentrations of each tocopherol. The coefficients of variation were <10% for tocopherols and control pools. It is reported for YALTA that the intraclass correlation coefficients (ratio of between-person variance to between- plus within-person variance) was 0.93 for α-tocopherol .

# Statistical Analyses.

Multivariable linear regressions were performed at study year 0 to determine the association between α-tocopherol and γ-tocopherol and FEV_1_, FVC and FEV_1_/FVC. Data were stratified by race or adjusted for race as indicated. Data were adjusted for the recruitment center, age, age^2^, height, height^2^, sex, and body mass index (BMI), smoking status and asthma status. Age^2^ is significant in year 0 and therefore was necessary for Tables 3-4 because participants <25 years old were kept in analysis as previously referred to by Hankinson et al . Height^2^ was also significant at year 0 and therefore adjusted as is consistent with data by Hankinson et al . Initially, α-tocopherol and γ-tocopherol were square-root transformed to reduce the mild skewness of the distributions, but statistical results in regression analyses using transformed data were consistent with results using the untransformed data. Therefore, all analyses utilized the untransformed tocopherol measures to simplify interpretation of beta results. The data were also analyzed with adjustment for waist circumference instead of BMI and the findings were similar; therefore the data with adjustment for BMI are presented in the tables. Adjustments are as listed in the tables and figures.

To examine the association of low and high levels of α-tocopherol or γ-tocopherol with spirometry parameters in non-asthmatic participants, participant data from year 0 were placed into a set of quartiles for serum α-tocopherol and a set of quartiles for serum γ-tocopherol. After the data were split into quartiles, we assessed two-way and three-way interactions of the tocopherol quartiles and race and adjusted for all the factors listed in the previous analysis, except asthma status. For FEV1 and FVC, the p-values for the three-way interactions were 0.11 and 0.14, respectively, but for FEV1/FVC the p-value was 0.56. Therefore, we stratified all analyses by race and conducted multivariable linear regressions within each quartile to determine (1) the associations of FEV­_1_ and FVC with γ-tocopherol within each quartile of α-tocopherol and (2) the associations of FEV­_1_ and FVC with α-tocopherol within each quartile of γ-tocopherol. Within each quartile, the data was analyzed using the SAS (Cary, NC, USA) GLM procedure. Due to significant interactions of race with α-tocopherol and with γ-tocopherol (p < 0.0001 in each case), data were stratified by race and adjusted for the recruitment center, age, age^2^, height, height^2^, sex, and body mass index (BMI), smoking status and asthma status. Age^2^ was included because participants <25 years old were kept in analysis.

To examine the association of γ-tocopherol with FEV­_1_, FVC and FEV­1/FVC as a function of participant age, we used generalized estimating equations (GEE). For GEE, we used spirometry data from study year 5, when the age range of the cohort was 21 to 35 years and when lung development is achieved, and study years 10, 20, and 25. The average tocopherol was from study year 0, 7 and 15. We defined four γ-tocopherol groups to model average Western European γ-tocopherol levels (1-2 µM γ-tocopherol) , moderate U.S.A. population γ-tocopherol levels (3-4.8 µM, the average published for NHANES in the U. S.A. ), moderate-high U.S.A. population γ-tocopherol levels (4.9-10 µM) and highly-elevated U.S. population γ-tocopherol levels (>10 µM).

We examined the 15-year trends in the year 0, 7, and 15 α-tocopherol and γ-tocopherol measures by fitting a random intercept and trend mixed regression model for each. The model for α-tocopherol indicated the year 0 intercept was 20.4 μM and the slope, which was highly significant (p<0.0001), was increasing at a rate of 0.42 μM/year. The covariance parameters (intercept variance=9.2330, slope variance=0.08840, intercept and slope covariance=0.2675) quantify the individual heterogeneity in the intercepts and slopes. These values yield an intercept STD=0.4827, slope STD=0.2973, and correlation between intercept and slope=0.296, indicating 95% of the participants had slopes in the interval (-0.1608, 1.0046) and participants with higher year 0 intercepts tended to have steeper increasing slopes. In other words, participants with high α-tocopherol at year 0 tended to remain high relative to other participants over the 15-year follow-up. Findings for γ-tocopherol were similar: the year 0 intercept was 5.00 μM and slope was increasing at 0.083 μM/year. Intercept and slope STDs were 1.0541 and 0.1058, respectively, and the intercept and slope correlation was 0.233. Because individuals tended to maintain their tocopherol relative positions over 15 years, we used each participant’s 15-year α-tocopherol and γ-tocopherol averages in subsequent longitudinal analyses of FEV1, FVC, and FEV1/FVC versus age. Therefore, for the longitudinal analysis of association of γ-tocopherol with spirometric parameters, we averaged the year 0, 7 and 15 γ-tocopherol concentrations for each participant. To limit the potential effects of very high α-tocopherol on lung function , it was determined a priori to choose participants with a serum α-tocopherol level less than 37.1 µM because it was two standard deviations above the mean concentration of serum α-tocopherol in the 20-39 year age group from the U.S. National Health and Nutrition Examination Survey (NHANES) . Therefore, after exclusions primarily for individuals that were missing tocopherol, spirometry or covariate data at follow-up years (n=623) and exclusion of a few individuals with very high serum α-tocopherol levels (over 37.1 µM) (n=146), there were 3757 participants for analysis.

Because our analysis of this CARDIA cohort revealed similar associations of α-tocopherol and γ-tocopherol in both races, we did not stratify the data by race in this analysis, but instead we adjusted for race to maintain the sample size of the highest and lowest γ-tocopherol groups. We used two statistical models. In the first model, for presentation in the figure, we categorized age into age groups and formulated FEV_1_, FVC and FEV_1_/FVC as a function of the following 7 age categories (ages 21-27, 28-31, 32-35, 36-39, 40-43, 44-47, 48-55). Categories are 4 year age ranges except the first and last categories which were slightly larger because there were fewer participants at these ages. Second, for assessing significance of associations, we modeled FEV_1_, FVC and FEV_1_/FVC as functions of age, treating age as a continuous variable centered at 23 years. For both GEE models, we included the following variables: center, race, exam year, height, height^2^, sex, BMI, smoking status, asthma group, average α-tocopherol concentration, 3 dummy variables representing the 4 γ-tocopherol groups, and the interactions of the four γ-tocopherol groups with age. BMI, height, and smoking status were analyzed as time-dependent covariates. This GEE analysis was also performed with percent predicted values for FEV_1_ and the statistical outcomes were the same (data not shown).

**Supplement References.**

1. Smith LJ, Arynchyn A, Kalhan R, Williams OD, Jensen R, Crapo R, Jacobs Jr DR. **Spirometry guidelines influence lung function results in a longitudinal study of young adults**. *Respir Med* 2010: **104**: 858-864.

2. Gross M, Yu X, Hannan P, Prouty C, Jacobs DR, Jr. **Lipid standardization of serum fat-soluble antioxidant concentrations: the YALTA study.** *Am J Clin Nutr* 2003: **77**(2): 458-466.

3. Bieri JG BE, Smith JC. **Determination of individual carotenoids in human plasma by high performance chromatography.** *J Liquid Chromatogr* 1985: **8**: 473-484.

4. Craft N, Brown E, Smith J, Jr. **Effects of storage and handling conditions on concentrations of individual carotenoids, retinol, and tocopherol in plasma**. *Clin Chem* 1988: **34**(1): 44-48.

5. Gross M, Prouty C, Jacobs D, Jr. **Stability of carotenoids and alpha-tocopherol during blood collection and processing procedures.** *Clin Chem* 1995: **41**(6): 943-944.

6. Iribarren C, Folsom AR, Jacobs DR, Jr., Gross MD, Belcher JD, Eckfeldt JH. **Association of serum vitamin levels, LDL susceptibility to oxidation, and autoantibodies against MDA-LDL with carotid atherosclerosis. A case-control study. The ARIC Study Investigators.** Atherosclerosis Risk in Communities. *Arterioscler Thromb Vasc Biol* 1997: **17**(6): 1171-1177.

7. Hankinson JL, Odencrantz JR, Fedan KB. **Spirometric reference values from a sample of the general U.S. population.** *Am J Respir Crit Care Med* 1999: **159**(1): 179-187.

8. Cook-Mills JM, McCary CA. **Isoforms of Vitamin E Differentially Regulate Inflammation.** *Endocr Metab Immune Disord Drug Targets* 2010: **10**: 348-366.

9. Hu G, Cassano PA. **Antioxidant Nutrients and Pulmonary Function: The Third National Health and Nutrition Examination Survey (NHANES III).** *Am J Epidemiol* 2000: **151**(10): 975-981.

10. McCary CA, Abdala-Valencia H, Berdnikovs S, Cook-Mills JM. **Supplemental and highly elevated tocopherol doses differentially regulate allergic inflammation: reversibility of alpha-tocopherol and gamma-tocopherol's effects**. *J Immunol* 2011: **186**(6): 3674-3685.
